# Modeling and Quantifying the Impact of Personified Communication on Purchase Behavior in Social Commerce

**DOI:** 10.3390/bs13080627

**Published:** 2023-07-28

**Authors:** Jie Zhao, Chengxiang Zhu

**Affiliations:** School of Business, Anhui University, Hefei 230601, China; m21201037@stu.ahu.edu.cn

**Keywords:** social commerce, social media, personified communication, e-commerce

## Abstract

The advancement of mobile internet technology has enabled companies to leverage social media for e-commerce, where some use personified images and language to communicate with consumers. This paper investigates how personified communication affects consumer behavior in social commerce and whether consumers are willing to accept this new form of communication. Specifically, the study explores consumers’ willingness to accept personified communication in social commerce, considering the role of cognitive needs in regulating the internal mechanism. The paper proposes suggestions for enterprises to improve their social media communication and presents an improved model based on the Technology Acceptance Model (TAM). The model introduces perceived interaction as a new independent variable and adds cognitive needs as a regulatory variable, which is more suitable for social commerce. The study conducts a questionnaire survey online and analyzes the data using AMOS and SPSS. The results demonstrate that perceived usefulness and perceived interaction positively impact consumers’ attitudes, which subsequently influences their willingness to purchase. Furthermore, cognitive needs as a regulatory variable significantly affect the path from perceived usefulness to attitude and purchase intention.

## 1. Introduction

With the development of Web 2.0 and mobile internet technologies, social commerce has emerged as a promising area [1,2,3]. Social media platforms provide a space where internet users can passively accept information generated by other users and communicate with others freely. Initially, social media was viewed as an entertainment tool. However, during the COVID-19 pandemic, some e-commerce providers discovered that social media could broaden the scope of information dissemination and social interaction [4]. One of the key advantages of social commerce is the ease of interaction, as users can conveniently engage with each other on social media platforms. Many brand companies open accounts on social media platforms, such as Sina Weibo, Twitter, and WeChat, and have started to offer personification services in social media. For example, “Haier”, a famous appliance supplier in China, has promoted constructing a “brand-consumer” relationship through personification communication in the WeChat platform. A recent empirical study conducted on 338 valid samples showed that personified communication positively impacted customer purchase behavior [5].

In recent years, the academic community began to pay attention to social commerce [6]. The research on social commerce mainly focused on the following aspects: the theoretical concept of social commerce, the marketing strategy of social media, the brand attitude of consumers, and so on. However, these studies have not yet focused on the impact of marketing on consumer psychology and behavior in the context of social media. Enterprises pay more and more attention to marketing activities under social media’s background, reflecting the innovation of brand communication and consumer interaction mode, and consumers’ behavior and psychology are bound to be affected by this innovation mode. Personified communication is an innovative behavior of enterprises in social commerce.

This paper constructs a research model of consumers’ willingness to accept personified communication with brand merchants in social commerce. This model aims to answer the question “whether the personified communication behavior of brand merchants has an impact on consumers, and if so, how it will be affected?”. Specifically, this paper proposes an improved model based on the TAM model, in which “perceived interaction” is introduced as an independent variable and “cognitive need” as a moderating variable, which is more in line with the characteristics of social commerce. On this basis, the paper obtains data through a questionnaire survey conducted on the WeChat platform and uses AMOS and SPSS for data analysis. This paper enriches the existing relevant theories of social commerce, enhances brand merchants’ attention on the innovation of social media marketing methods, and provides corresponding decision-making suggestions for companies to carry out E-commerce on social media platforms.

Note that a number of models have been proposed to explain user acceptance of new technologies, and different factors were introduced to measure the impact on user behavior. Among all existing models, TAM [7] and UTAUT [8] have been demonstrated to have high explanatory power for user acceptance of new information technologies [9]. Although the UTAUT model provides a unified view based on eight models, including TAM, it is mainly proposed for corporate-level analysis. On the contrary, TAM emphasizes individual-level analysis. In this paper, we adopt the TAM model as the basic research model. There are two reasons. First, this study focuses on personified communication, primarily reflecting individual users’ feedback. Thus, we believe TAM is more suitable than UTAUT for this study because TAM is based on an individual level. Second, many studies in social commerce have applied the TAM model to analyze the user acceptance of online social behavior. The research scope of this paper, i.e., personified communication, has the characteristics of both online media and social networks; thus, it is a reasonable choice to select the TAM model. On the other hand, both TAM and UTAUT lack factors reflecting user interaction, which is the most influential factor in personified communication. Thus, differing from the traditional TAM model, we propose a new construct, “perceived interaction”, and a new regulatory variable, “cognitive need”, to enhance the TAM model, making the model more suitable for studying the impact of personified communication on purchase behavior in social commerce.

The rest of the paper is structured as follows. In Section 2, we briefly describe the related work of this study. Section 3 gives the research design. Section 4 presents data analysis and empirical results. Finally, in Section 5, we conclude the entire paper and present some useful suggestions.

## 2. Related Work

### 2.1. Social Commerce

Social commerce refers to using social media to promote E-commerce, such as blogs, social tools, and sharing forums. Hammond pointed out that, unlike online marketing, the key to social commerce lies in creating and exchanging user content [10]. The research on social commerce mainly focuses on theoretical concepts, marketing strategy, brand attitude, etc. Scholars of theoretical concepts focus on the differences between social commerce and traditional marketing. For example, Chikandiwa reported that social commerce promotes interactive communication between users compared with conventional marketing’s one-way communication channel [11]. In social media, enterprises should transmit information to consumers and accept consumers’ ideas and opinions. Also, Jung et al. pointed out that social commerce can promote advertising at a lower cost and establish stable user relationships [12]. Kiezmann et al. developed a cellular model to facilitate enterprises’ social media use for marketing positioning regarding marketing strategy research [13]. Their model includes seven modules: identity, communication, sharing, existence, relationship, reputation, and aggregation.

On the other hand, Mohammadia proposed using social media to publish information using the same language style as consumers [14]. Their study showed that transparent information was more conducive to marketing. Wawrowski and Otola studied social commerce in creative industries and proposed to use social commerce to promote computer games [15]. Hudson et al. pointed out that social media communication significantly affects corporate brand image. Social media communication has a more significant impact on corporate brand image than conventional media communication and is more suitable for improving brand image [16].

On the one hand, the existing research clearly defines the types of social commerce and points out the differences between traditional marketing and social commerce. On the other hand, it also explores social media strategies. Although social commerce has attracted Chinese scholars’ attention, there is still a lack of relevant research. There is not enough attention on enterprises’ marketing methods using social media. A complete system has not yet been presented.

### 2.2. Personified Communication

Personified communication is an innovative way for companies to use social media to offer a personified communication mode to interact with users [17,18]. It is an innovative way for brand companies to use social media for marketing. Previous research on personification mainly focused on the design concept of an entity. For example, Aaker pointed out that marketers visualize, vividly, and personify products when facing young children to make them understand [17]. Simultaneously, the design concept of personification has also been integrated into the automobile industry. For example, in the Super Bowl in 2007, General Motors adopted personified cars. With social media development, personification is not limited to traditional appearance design but gradually transformed into online communication. Social media has provided a platform for companies to use personified communication for marketing. Salminen et al. conducted an empirical study and found that companies can improve consumers’ attitudes through personified advertisements [18]. Nobile and Kalbaska established a platform for companies to use personified communication [19]. The relationship between consumers’ belonging needs and brand attitude was explored by setting up a personified situation. In addition, Puzakoza et al. found that consumers’ personal beliefs moderate personified communication [20].

Personified communication has just entered the vision of managers and researchers under the social network background, which belongs to a relatively new concept. As a result, most of the current literature research focuses on theoretical concepts and brand attitudes. Although many well-known brands use social media to conduct personified communication with consumers and achieve good marketing results, how they affect consumer behavior is still unclear.

### 2.3. Technology Adoption Model (TAM) and Beyond

The Technology Acceptance Model (TAM), which is an adaptation of the Theory of Reasoned Action (TRA) [21], was developed to describe users’ behavior to accept or reject the use of new technologies [7]. TAM aims to explain the determinants of user acceptance of information technologies, which can explain user behavior towards a range of information technologies. TAM defines two variables, namely perceived usefulness and perceived ease of use, to quantify user attitude to information technology, which can be used to measure user acceptance of information technologies. Although the TAM model was initially designed to explain and predict the behavior of individuals on the use of information systems, it has been used in many other studies [22,23,24].

The UTAUT (Unified Theory of Acceptance and Use of Technology) model [8] is also a technology acceptance model developed and reviewed based on eight earlier models, including TRA and TAM. To this end, TAM can be regarded as a sub-system of UTAUT [25,26]. UTAUT holds that there are four fundamental constructs: (1) performance expectancy, (2) effort expectancy, (3) social influence, and (4) enabling conditions. The initial three are determinants of usage intention and behavior, while the fourth is a determinant of user behavior. It should be acknowledged that the initial UTAUT model was established in order to determine the implementation and usage of technologies in a corporate sense [27].

There are also other research models for measuring user purchase intentions, such as IAM (Information Adoption Model) [28], UTAUT2 [29], SDT (Self-Determination Theory) [30], and SOR (Stimulus-0rganism-Response) [31]. So far, according to a recent survey, the TAM model and UTAUT model are two key models that have been widely adopted by many existing studies [9]. Although the UTAUT model provides a unified view based on eight models, including TAM, it is mainly proposed for corporate-level analysis. On the contrary, TAM emphasizes individual-level analysis.

This paper also adopts the TAM model as the basic research model. It should be noted that both TAM and UTAUT lack considering the impact of user interaction, which is the most influential factor in personified communication. Thus, we propose a new construct, “perceived interaction”, and a new regulatory variable, “cognitive need”, to enhance the TAM model, making the model more suitable for studying the impact of personified communication on purchase behavior in social commerce.

## 3. Research Method

### 3.1. Hypothesis

#### 3.1.1. Hypothesis on the Influence of Consumers’ Attitude on Purchase Intention

Ajzen has pointed out that people’s behavior and attitude will directly affect their intention [32]. Scholars have made a clear definition of attitude and behavior. Behavioral attitude refers to a person’s positive or negative feelings and cognition of specific behavior, which is a person’s view of the behavior after evaluating its total value. Behavioral intention refers to an individual’s willingness and intensity to a particular behavior. The more robust the willingness is, the more significant the behavior’s possibility will be. The theory of rational behavior points out that people’s behavior attitude affects the generation of behavior intention, affecting action implementation.

Davis proposed the technology acceptance model (TAM) [7] based on the above theory, which explores people’s behavior and attitude toward its acceptance. This paper improves the TAM model for brand merchants’ personified communication behavior. Brand merchants adopt personified communication behavior in social media to influence consumers’ purchase intention. Based on the TRA theory [21], consumers’ purchase intention is determined by their purchase attitude. Therefore, this paper puts forward the following assumptions:

**H1:**  *The attitude of consumers’ purchase behavior positively impacts consumers’ purchase intention.*

#### 3.1.2. Hypothesis on the Influencing Factors of Consumers’ Attitude

The TAM model defined perceived usefulness as an indicator. According to its definition, combined with personified communication, we define perceived usefulness as consumers’ perception of life benefits when they receive personified communication information from brand merchants in social media. Perceived usefulness is an important indicator of consumer evaluation. Perceived usefulness can evaluate the benefits of behavior, and perceived cost can assess the level of behavior cost.

In personified communication, perceived usefulness can make consumers perceive the quality of products; if consumers can feel the usefulness of personified communication behavior in social media, they will have a positive attitude toward purchasing behavior [18]. Based on this, this paper puts forward the following assumptions:

**H2a:**  *Perceived usefulness has a positive impact on consumers’ purchase attitudes.*

Chikandiwa et al. pointed out that enterprises use social media for marketing activities because, compared with traditional marketing methods, social commerce is more interactive and can effectively establish consumer-brand relationships, positively impacting users’ purchase attitudes [11]. Additionally, consumer participation is an essential factor in the success of enterprises using social media. Consumers’ involvement in enterprise marketing activities is also conducive to consumers’ integration into brand companies.

Consumers’ participation is crucial when enterprises adopt personified communication behavior through social commerce. Interaction is a key feature in social commerce, influencing users’ purchase attitudes [33]. Through the activity analysis, we found that the personified communication of enterprises mainly includes two aspects of interaction. One is the interaction between companies and users, called User-Company interaction in this paper. Through this level of interaction, the needs of consumers can be understood by enterprises to improve products, and consumers can also obtain information directly from enterprises, which strengthens communication with enterprises and establishes enterprises well. The other is user interaction, which is called User-User interaction in this paper. It can promote consumers’ trust and promote the formation of word-of-mouth. Based on this, this paper puts forward the following assumptions:

**H2b:**  *User-Company interaction has a positive impact on consumers’ purchase attitudes.*

**H2c:**  *User-User interaction has a positive impact on consumers’ purchase attitudes.*

**H3a:**  *Consumers’ purchase attitude mediates the relationship between perceived usefulness and purchase intention.*

**H3b:**  *Consumers’ purchase attitude mediates the relationship between perceived interaction (User-User) and purchase intention.*

**H3c:**  *Consumers’ purchase attitude mediates the relationship between perceived interaction (User-Company) and purchase intention.*

#### 3.1.3. Hypothesis of Consumers’ Cognitive Need

In the personified communication marketing mode in social media, consumer psychological perception changes depending on enterprise behavior and consumer characteristics. Consumers’ personality characteristics are different, and their attitudes toward enterprise marketing differ from taking other actions. Cognitive need refers to how individuals participate in thinking and enjoy thinking in the process of understanding things; consumers with high cognitive needs are eager to obtain more product information, which can help them make purchase decisions [34]. People with low cognitive needs desire to make decisions as soon as possible. According to cognition, consumers with high cognitive needs are eager to obtain more information about product attributes to help them make decisions as soon as possible. People can be divided into two categories: those with low cognitive needs think the situation should be orderly and regular.

In contrast, those with high cognitive needs will analyze the situation according to their experience and understand it through self-learning [34]. Ma et al. pointed out that people with high cognitive prefer to form brand attitudes through thinking [35]. Conversely, people with low cognitive needs are easily affected by the frontier clues of advertising.

When the consumer’s cognitive need is high, even if the company’s personified information provides the product’s detailed content, such consumers are eager to obtain more additional information to determine their purchase behavior. Therefore, even if the perceived usefulness is high, consumers with higher cognitive needs still want to get more information than those with low cognitive needs. The change in their purchasing attitude can be improved. It can be relatively slow. Consumers with low cognitive needs may promote their decision-making through interactive communication when the interaction is high. Therefore, the higher the perceived interaction, the more pronounced the change in consumers’ attitude towards cognitive needs; however, when consumers with high cognitive needs desire to make self-determination, they may perceive that interaction has little effect on their decision-making. Based on this, this paper puts forward the following assumptions:

**H4a:**  *Cognitive needs moderated the relationship between perceived usefulness and attitude; when cognitive needs were high, perceived usefulness had a less positive effect on attitude.*

**H4b:**  *Cognitive needs have a moderating effect on perceived interaction and attitude relationship; when cognitive need is high, perceived interaction has a less positive impact on attitude.*

### 3.2. Research Model

**Figure 1:**
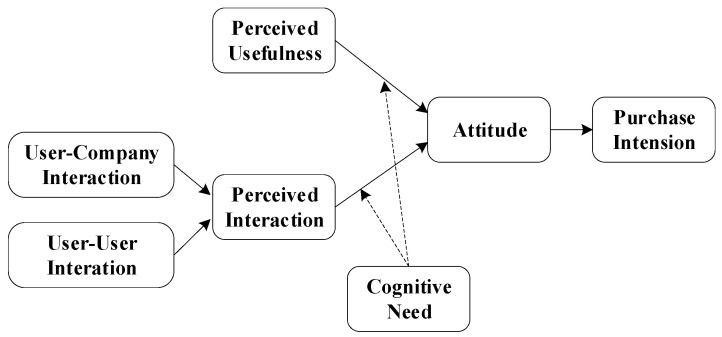
Research model.

Based on the technology acceptance model (TAM), this paper constructs a model of consumers’ willingness to accept personified communication with brand merchants. The TAM model is revised based on the rational behavior theory, which believes that people’s behavior intention is affected by their behavior attitude. The TAM model is widely used in various fields to explore users’ willingness to accept technology or similar technology. Personified communication is an innovative marketing method adopted by companies. Consumers’ willingness to accept companies’ marketing methods can be measured by their desire to buy. In brand companies, the perceived ease of use and perceived usefulness of information will affect consumers’ purchase attitudes, affecting their purchase intention. In the personified communication of companies, perceived interaction is an essential factor influencing the attitude of users. Therefore, with the introduction of perceived interaction, the TAM model can study consumers’ acceptance intention of personified communication behavior. Figure 1 shows the theoretical model of this paper.

## 4. Empirical Study and Results

### 4.1. Questionnaire Design

This paper conducts a questionnaire survey to test the model hypothesis. All subjects gave their informed consent for inclusion before they participated in the study. The study was conducted in accordance with the Declaration of Helsinki, and the protocol was approved by the Ethics Committee of the Anhui Philosophy and Social Science Foundation (AHSKY2021D15).

The questionnaire is designed according to the general principles and steps of the previous literature. We extracted each variable’s indicators and tested the designed questionnaire in a small range of 50 college students based on many existing studies. The 50 students were randomly invited through internal WeChat groups established at Anhui University. Note that this surely makes the samples of this study younger than other studies. However, since personified communication is a new IT-enabled thing that is mostly used by young people, the sampling of users is still meaningful to the study. According to the analysis results of the sample, we revised some items to get the formal questionnaire. The questionnaire was designed with the Richter five subscale, which has been adopted by many previous studies [22,24]. The respondents chose 1 (very disagree) to 5 (very agree) to rate the questions. The scale of perceived usefulness comes from the research in [36]. The scale of perceived interaction is designed according to the study in [37,38]. The scale of purchase attitude is developed according to [39,40]. The scale of cognitive needs was modified based on the scale designed [34]. The questionnaire design is shown in Table 1.

### 4.2. Data Collection

The data collection of this paper is mainly through the WeChat platform, the biggest personal communication platform in China. We use WenJuanXing (https://www.wjx.cn/, accessed on 1 April 2023) to prepare questionnaires and collect data. WenJuanXing is China’s biggest online survey platform, which also provides apps for various mobile operating systems like Android and IOS. We generate the questionnaire link using the WenJuanXing app on the WeChat platform and share the link with different user groups. Then, all questionnaire data will be collected into a uniform file supported by WenJuanXing. To ensure that participants have a good understanding of personified communication and provide real, accurate, and effective data, they were asked some questions before starting the survey, such as “Do you often know about products or after-sales service information through retailers’ social media?” and “Are you familiar with other social commerce models?”. Only those who responded positively were invited to fill in the questionnaire. In this survey, 340 questionnaires were collected. After eliminating the invalid questionnaires, such as no experience in using social media and lack of data, 252 valid questionnaires were obtained, with an effective rate of 74.1%. The sample size is more than 200, which meets the analysis requirements of the structural equation model. Among the respondents, 127 are male, 125 are female, the age is mainly between 20 and 25 years old (72.2%), and 68.6% have a college degree or above. Every user has experience in using social networks. 172 (68.2%) users have touched personified communication through enterprise microblogs, and 110 (43.7%) users have experiences of social commerce, i.e., performing online shopping via enterprise social-network platforms.

### 4.3. Model Validation

The model validation measures the relationship between latent variables and measurement indicators. We use confirmatory factor analysis (CFA) to analyze its reliability and validity.

(1) Reliability. We use SPSS 22.0 to analyze reliability. The specific results were KMO = 0.874. The Bartlett sphericity test results were significant (SIG = 0.000). The Cronbach’s α coefficient and combination reliability of the structural variables were greater than 0.8, indicating the scale’s high reliability. The detailed results are shown in Table 2;
(2) Convergence validity. The standardized factor loads of the significant variables of the model were higher than 0.8 and reached a considerable level. The model’s component reliability was more significant than 0.7. The average variance extraction rate was more significant than 0.5;
(3) Discriminant validity. As shown in Table 3, each variable’s correlation coefficient is less than the square root of the average variance extraction rate of the corresponding variable, so we can know that it has good discriminant validity.

### 4.4. SEM Analysis

**Figure 2:**
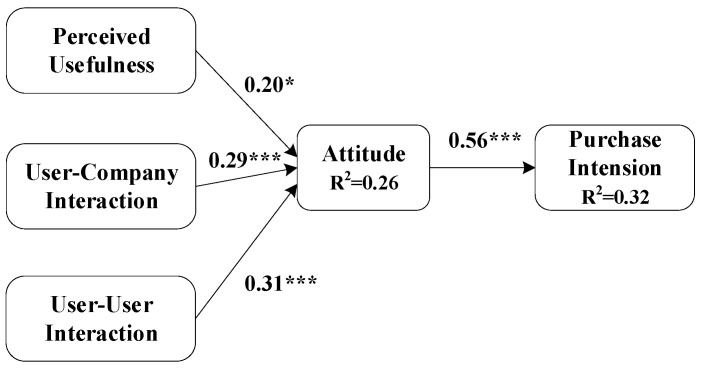
SEM test design (*** denotes *p* < 0.001, * indicates *p* < 0.05; R^2^ is the decisive coefficient).

The structural equation model (SEM) measures the relationship between latent variables. We use AMOS 23.0 to verify the path coefficients among the latent variables in the research model. The path coefficients among the variables are shown in Figure 2.

Companies’ personified marketing affects consumers’ attitudes through perceived usefulness and interaction when consumers browse on social media. More specifically, perceived usefulness significantly affects consumers’ attitudes (β = 0.20, *p* < 0.05), and perceived interactive consumers also significantly and positively affect consumers’ attitudes (β = 0.29, *p* < 0.001). The results show that the change in knowledge interactive enterprises’ attitude to consumers is also positively significant (β = 0.31, *p* < 0.001). Thus, hypotheses H2a, H2b, and H2c are valid. This result shows that when companies adopt personified communication in social commerce, perceived usefulness and interaction positively affect users’ purchasing behavior.

On the other hand, the personified communication marketing methods adopted by enterprises significantly impact users’ purchase behavior. When consumers’ attitudes toward purchase behavior change, the impact on purchase intention is positive and significant (β = 0.56, *p* < 0.001), consistent with the rational behavior theory. The rational behavior theory points out that people’s attitudes to behavior will significantly impact behavior intention, affecting the implementation of behavior. In other words, the change in consumers’ purchase attitudes will positively impact the change in purchase intention.

Also, the determinable coefficient of attitude is 0.26, showing that perceived usefulness and perceived interaction explain the variance of 26% of consumers’ attitudes towards purchase behavior when browsing social media and experiencing personification. Overall, the decisive coefficient of purchase intention is 0.32, which means that the model explains a 32% variance variation of consumers’ purchase intention, and the explanation degree is acceptable. The model fitting degree is shown in Table 4. The model fit index reaches the theoretical value, and the fit degree is sufficient.

### 4.5. Mediating Effect Test

To verify the influence of personified communication on consumers’ purchase intention, we adopt the method of Hayes and Scharkow [41] to analyze the mediating effect of variables. The results are shown in Table 5.

To sum up, in the path of perceived usefulness influencing consumers’ purchase intention, the attitude has a partial mediating role, which shows that perceived usefulness mainly affects consumers’ attitude towards purchase intention and then affects consumers’ willingness to purchase behavior. In the path of consumers’ perceived interaction influencing consumers’ purchase intention, the mediating effect of attitude on consumers’ perceived interaction on consumers’ purchase intention exists. Unlike the other two paths, attitude partially influences perceived interaction at the enterprise level on consumers’ purchase intention. Thus, consumers’ purchase attitude mediates the relationship between perceived usefulness and purchase intention, validating H3a. Furthermore, consumers’ purchase attitude mediates the relationship between perceived interaction (User-User) and purchase intention, supporting H3b. Finally, consumers’ purchase attitude mediates the relationship between perceived interaction (User-Company) and purchase intention, meaning that H3c is established. These results show that perceived usefulness and perceived interaction impact purchase intention through the influence of attitude.

### 4.6. Moderated Mediation Analysis

In this study, the condition’s indirect effect under different variables’ values is directly obtained by the Process operation. The process will automatically operate different values to reduce one standard deviation and increase one standard deviation based on adjusting the variable’s mean value from low to high [42]. The results shown in the left part of Table 6 shows that when consumers’ cognitive needs are relatively low, the indirect effect of the perceived usefulness of personified communication on consumers’ purchase intention is 0.067. When consumers’ cognitive needs are relatively high, the indirect effect of perceived usefulness on consumers’ purchase intention through attitude is 0.125. Since these confidence intervals do not contain zero, the results show that the indirect effect of perceived usefulness on the dependent variable consumers’ purchase intention through attitude is significant whether the moderator of cognitive need is low or high. Additionally, when the cognitive need is low, the perceived interaction’s indirect effect on consumers’ purchase intention is 0.033. When the consumer’s cognitive need is relatively high, the indirect effect of perceived interaction (User–User) on consumers’ purchase intention through attitude is 0.057.

Similarly, the indirect effect of perceived usefulness on consumers’ purchase intention through attitude toward the dependent variable is significant. In the aspect of perceived interaction (User–Company), when consumers’ cognitive need is low, the indirect effect of perceived interaction on consumers’ purchase intention through attitude is 0.109 (the confidence interval is [0.042,0.215]). On the other hand, when the cognitive need is relatively high, the indirect effect is 0.111 (the confidence interval is [0.012,0.239]). Therefore, the perceived interaction (User–Company) significantly impacts purchase intention through attitude.

According to the above analysis, we can see that it is not enough to determine whether there is a moderated mediating effect by only conducting the study of the conditions’ indirect impact. Therefore, the right part of Table 6 focuses on the index obtained from the process operation. We can see that in the path: PU→AT→PW, the judgment index of cognitive need on the indirect relationship between perceived interaction of personified communication and consumer purchase intention is 0.0642 (the confidence interval is [0.023,0.0127]). Therefore, the moderated mediating effect is significant since the confidence interval does not contain zero. This result fully supports hypothesis H4a.

Regarding perceived interaction (User–User), the judgment index for the moderating effect of cognitive need on the indirect relationship between perceived interaction and purchase intention is 0.0132. Regarding perceived interaction (User–Company), the moderating effect of cognitive need on the indirect relationship between perceived interaction and purchase intention is 0.0016. As the confidence interval contains zero, the mediating effect is insignificant, and hypothesis H4b has not been verified.

## 5. Conclusions and Discussion

### 5.1. Research Conclusions

Based on the TAM theory, this paper studies the influence of personified communication on consumers’ purchase intention under the background of social commerce. The results show that personified communication significantly impacts consumers’ attitudes through perceived usefulness between enterprises and consumers. Such results are consistent with previous studies based on TAM [5,17,18,19,20]. Also, we find that personified communication positively impacts users’ purchase attitudes through perceived interaction, which is a new finding of this study. Furthermore, the mediating effect test shows that consumers’ psychological perception indirectly affects consumers’ purchase intention through attitude. Specifically, consumer psychological perception (perceived usefulness, perceived interaction) affects consumers’ purchase intention by influencing consumers’ attitudes. Due to the anonymity, spatial separation, and the characteristics of online products, in the context of social media, when users browse online information, they meet the personified communication behavior of enterprises to publicize their products. To judge the authenticity of the information, they long for enterprises’ information to be authentic and reliable, reduce uncertainty, and perceive the use-value of products. Furthermore, users can obtain additional information by communicating with enterprises and other consumers in the background. Therefore, the more perfect the information provided by personified communication is, the higher the perceived usefulness is, and the more significant the positive impact on attitude is.

In addition, the influence of attitude on purchase intention is also substantial, consistent with previous studies reporting that attitude positively affects behavioral intention [1,2,3,4,5,6,7,8,9,10,11,12,13,14,15,16,17,18,19,20,22,24]. In addition, consumers’ cognitive need for knowledge can adjust the path of perceived usefulness, indirectly influencing consumers’ purchase intention. In contrast, the indirect effect of perceived interaction on consumers’ purchase intention is insignificant. This shows that consumers’ cognitive needs may be satisfied after reducing uncertainty through interaction in online shopping, so the moderating effect is insignificant.

### 5.2. Suggestions

#### 5.2.1. E-Commerce Providers Are Suggested to Offer High-Quality Service Information through Social Media

The perceived usefulness of personified communication positively impacts consumers’ attitudes and then on consumers’ purchase intentions. The quality of the information provided by personified communication affects consumers’ perceived usefulness. Based on this, enterprises can bring novelty and entertainment to consumers through personified communication. They can also report information about commodity attributes, such as price, size, and material, in personified language. In this way, consumers’ perceived usefulness can be significantly improved, positively affecting consumers’ attitudes toward purchasing behavior. On the other hand, if personified communication only uses corporate brand cartoon characters to communicate with consumers, and the information about commodities is vague, it may not bring consumers a sense of usefulness.

#### 5.2.2. E-Commerce Companies Must Communicate Better with Consumers through Social Media

Due to social media’s rapid development, consumers can comment on and praise the information released by enterprises through social media. For these interactive behaviors of consumers, enterprises should take corresponding positive response behavior in time. Enterprises can interact in time to effectively improve the attitude of consumers to purchase behavior [43]. To improve consumers’ purchase intention, enterprises must take timely measures to influence the negative attitude positively.

#### 5.2.3. E-Commerce Providers Are Encouraged to Promote Consumer Interaction on Social Networks

The perceived interaction between users can indirectly affect consumers’ purchase intention through attitude [44]. Additionally, consumers are more willing to believe information sharing than enterprises’ information. The information enterprises provide must be confirmed by consumers, further affecting consumers’ purchase intentions. Therefore, enterprises should encourage interaction between consumers and reward excellent respondents in personified communication in social media, yielding brand co-creation of e-commerce providers and consumers [45].

#### 5.2.4. E-Commerce Companies Are Suggested to Create Topics in Social Media and Offer Rewards to Interactive Users

Creating topics and rewarding prizes can effectively guide consumers to interact with content, direction, and quantity. According to previous studies, reviews positively impact consumers’ purchase intention. The more quality reviews, potential consumers will know more product-related information and better perceive the product. If there are a lot of high-quality communication comments, it will attract more attention from consumers. The platform rewards the consumers who publish valuable and high-quality comments after reading the personified communication microblog information and gives material rewards, effectively improving consumers’ perception of interaction and usefulness and improving purchase intention.

### 5.3. Limitations and Future Work

There are a few limitations of this study. First, currently, we only improve the TAM model to form the research model for analyzing the impact of personified communication on users’ purchase behavior in social commerce. Although the research community has widely adopted TAM, it still has some drawbacks. Therefore, it is worthwhile to consider other research models in the future. Second, the questionnaires were collected through a Chinese social platform, making the research results of this study not totally applicable to other countries. In addition, the current users are restricted to the students in the university. To this end, this study is especially meaningful for young people.

The future work of this study will concentrate on the following aspects. First, we will consider integrating different research models, such as TAM and SOR, to develop a new research model with high explanatory power. Second, we will investigate the causality among constructs, making the research results more meaningful and explainable. Third, only interaction factors are added to the TAM model in our current model. In the future, we will consider other variables that may add more value to the research model, such as emotion [46] and trust [24]. Finally, the impact of personified communication on social commerce based on other social-network platforms, as well as on different user groups, is worth studying, which can make the proposed research model and the research results more convincing. However, as it is currently hard to access Twitter and Facebook in China, we will first consider other Chinese social networks, e.g., Sina Weibo, in the future.

## Figures and Tables

**Table 1:** Questionnaire questions and variables.

Variable	Item	Questionnaire Question
Perceived Usefulness (PU)	PU1	Reading about the personification of brand enterprises on Weibo has provided me with an opportunity to purchase my desired products.
	PU2	Reading about the personification of brand enterprises on Weibo has helped me to understand their products or services better.
	PU3	Reading about the personification of brand enterprises on Weibo has improved my shopping efficiency.
Perceived Interaction (PIU) (User-User)	PIU1	Through personified communication with enterprises, I have been able to increase the frequency of my interactions with other consumers.
	PIU2	Through personification communication with enterprises, I feel that my connections with other consumers have been strengthened.
	PIU3	As a consumer, I often engage in communication and interactions on the personified Weibo accounts of enterprises.
Perceived Interaction (PIC) (User-Company)	PIC1	When I post on Weibo about brand enterprises, I receive personified responses from the brand enterprise accounts.
	PIC2	I frequently participate in discussions on Weibo related to enterprise brands.
	PIC3	Through personification communication with enterprises, I feel that I am able to establish a connection with the enterprise.
Attitude (AT)	AT1	I am highly satisfied with the personified communication behavior of enterprises.
	AT2	The personified communication of enterprises has created an impulse for me to purchase their products.
	AT3	I believe that it is a good idea for enterprises to engage in personified communication on social media.
Purchase Intention (PW)	PW1	Personified communication by enterprises has been helpful for me in making purchasing decisions for their products.
	PW2	Personified communication by enterprises has influenced my decision to purchase products from this particular enterprise.
	PW3	When deciding whether to purchase a product, I consider the personified communication behavior of the enterprise.
	PW4	When I decide to purchase products from a brand, the personification communication behavior of the enterprise gives me more confidence in my decision.
Cognitive Need (CN)	CN1	I am eager to learn more about the products I want to purchase.
	CN2	I have a strong interest in the products that I purchase.
	CN3	I enjoy tackling problems that require a lot of thought.

**Table 2:** Reliability and validity test.

Variable	Item	Factor Loading	Cronbach’s α	CR	AVE
Perceived Usefulness (PU)	PU1	0.84	0.879	0.881	0.712
	PU2	0.85			
	PU3	0.84			
Perceived Interaction (PIU) (User–User)	PIU1	0.75	0.862	0.864	0.681
	PIU2	0.88			
	PIU3	0.84			
Perceived Interaction (PIC) (User–Company)	PIC1	0.83	0.896	0.898	0.746
	PIC2	0.89			
	PIC3	0.87			
Attitude (AT)	AT1	0.75	0.813	0.807	0.583
	AT2	0.79			
	AT3	0.75			
Purchase Intention (PW)	PW1	0.74	0.852	0.858	0.603
	PW2	0.74			
	PW3	0.84			
	PW4	0.78			
Cognitive Need (CN)	CN1	0.84	0.842	0.852	0.592
	CN2	0.80			
	CN3	0.71			

**Table 3:** Discriminant validity test.

	PU	PIU	PIC	AT	PW	CN
Perceived Usefulness (PU)	0.843					
Perceived Interaction (PIU) (User–User)	0.161	0.825				
Perceived Interaction (PIC) (User-Company)	0.071	0.034	0.863			
Attitude (AT)	0.191	0.245	0.226	0.763		
Purchase Intention (PW)	0.231	0.256	0.198	0.278	0.769	
Cognitive Need (CN)	0.230	0.257	0.266	0.373	0.335	0.875

**Table 4:** Model fitting degree.

Statistical Test	χ^2^/df	SMRM	RMSEA	AGFI	NFI	RFI	CFI	IFI	PGFI	PNFI	PCFI
Ideal Value	<2.00	<0.08	<0.05	>0.80	>0.90	>0.90	>0.90	>0.90	>0.50	>0.50	>0.50
Acceptable Value	<3.00	<0.1	<0.08	>0.70	>0.80	>0.80	>0.80	>0.80			
Our Value	1.46	0.04	0.04	0.91	0.94	0.92	0.97	0.98	0.67	0.76	0.79

**Table 5:** Mediating effects analysis.

Path	Effect	Bootstrapping (5000 Samples)				Result
		Bias-Corrected		Percentile		
		95% CI		95% CI		
		Lower	Upper	Lower	Upper	
PU→AT→PW	total	0.170	0.452	0.170	0.450	Exist
	direct	0.049	0.261	0.049	0.264	Exist
	indirect	0.019	0.318	0.010	0.311	Exist
PIU→AT→PW	total	0.205	0.530	0.197	0.515	Exist
	direct	0.014	0.250	0.001	0.237	Exist
	indirect	0.084	0.403	0.082	0.401	Exist
PIC→AT→PW	total	0.194	0.494	0.197	0.496	Exist
	direct	0.115	0.389	0.096	0.369	Exist
	indirect	−0.059	0.268	−0.044	0.285	Not Exist

**Table 6:** Moderated mediation analysis.

Path	Conditional Indirect Effect					Moderated Mediating Effect			
	Variable	Effect	Standard Error	Lower Bound	Upper Bound	INDEX	Standard Error	Lower Bound	Upper Bound
PU→AT →PW	Low	0.07	0.04	0.003	0.150	0.064	0.03	0.024	0.127
	High	0.12	0.04	0.045	0.218				
PIU→AT →PW	Low	0.03	0.06	0.022	0.150	0.013	0.04	−0.069	0.011
	High	0.06	006	0.042	0.187				
PIC→AT →PW	Low	0.11	0.04	0.042	0.215	0.002	0.05	−0.096	0.089
	High	0.11	0.06	0.012	0.239				

## Data Availability

Data sharing is not applicable to this article.
